# Results from Cardiovascular Examination Do Not Predict Cerebrovascular Macroangiopathy: Data from a Prospective, Bicentric Cohort Study

**DOI:** 10.3390/jcm14072366

**Published:** 2025-03-29

**Authors:** Johanna Lepek, Michael Linnebank, Lars Bansemir, Axel Kloppe

**Affiliations:** 1 Medical Clinic, Knappschaftskrankenhaus Bochum, 44892 Bochum, Germany; 2 Faculty of Health, University Witten/Herdecke, 58455 Witten, Germany; michael.linnebank@uni-wh.de; 3 Deptartment of Cardiology, Klinikum Niederberg, 42549 Velbert, Germany; lars.bansemir@helios-gesundheit.de; 4 Department of Cardiology, Angiology and Intensive Care Medicine, Marienhospital, 45886 Gelsenkirchen, Germany; a.kloppe@st-augustinus.eu

**Keywords:** coronary artery disease, cardio-CT, coronary angiography, cerebrovascular macroangiopathy, carotid stenosis, prognosis

## Abstract

**Background:** There is a large overlap in the risk profiles and pathophysiologies of coronary artery disease (CAD) and cerebrovascular macroangiopathy. Therefore, this study aimed to analyse whether findings in CAD examination by coronary angiography or cardio-computer tomography (cardio-CT) are predictive of cerebrovascular macroangiopathy. **Methods:** Our study was a prospective, bicentric, cross-sectional cohort study. A total of 191 patients without earlier CAD diagnosis who underwent a cardio-CT scan or coronary angiography for the screening of CAD during clinical routine were serially included. Two groups were formed based on the criterion of CAD (yes/no), and both were subsequently examined using sonography of the carotids. The CAD scores Syntax score I, Agatston equivalent score, and CAD-RADS score as well as AHA classification were determined. In cerebrovascular examinations, plaques and stenoses of the internal carotid artery (ICA) and the intima-media thickness (IMT) of the common carotid artery were analysed. Demographic and medical data such as the presence of arterial hypertension, diabetes mellitus, obesity, nicotine abuse, and dyslipidaemia were documented. The primary endpoint was the nominal association between CAD and ICA stenosis controlled for age and gender; secondary endpoints were correlations between ICA stenoses and CAD scores. **Results:** Of the 191 serially recruited patients (58% male, 65 ± 11 yrs.), 101 fulfilled CAD criteria; 90 did not. Of all patients, 137 had ICA plaques, and 11 thereof had an ICA stenosis ≥ 50%. No association was found between CAD and ICA stenosis (Wald = 0.24; *p* = 0.624). Accordingly, there was no association between IMT and Syntax score I (Wald = 0.38; *p* = 0.706), Agatston equivalent score (Wald = 0.89; *p* = 0.380), CAD-RADS score (Wald = 0.90; *p* = 0.377), or AHA classification (Wald = 0.21; *p* = 0.837). Common cardiovascular risk factors, i.e., arterial hypertension (Wald = 4.47; *p* = 0.034), diabetes mellitus (Wald = 7.61; *p* = 0.006), and nicotine abuse (Wald = 0.83; *p* = 0.028), were associated with newly diagnosed CAD but not with ICA plaques, stenosis, or increased IMT. **Conclusions:** In our cohort, newly diagnosed CAD was associated with typical risk factors. However, neither CAD nor these risk factors were associated with cerebrovascular disease. This suggests that in patients without prior CAD diagnosis, findings from CAD examinations might not be reliably predictive of cerebrovascular disease.

## 1. Introduction

There is a large overlap in the risk profiles and pathophysiologies of coronary artery disease (CAD) and cerebrovascular disease (CVD). This study aimed to analyse whether coronary angiography or cardio-computer tomography (cardio-CT) findings are predictive of cerebrovascular disease such as plaques and stenoses of the internal carotid artery (ICA) or increases of the intima-media-thickness (IMT) of the common carotid artery (CCA).

Both, CAD and CVD, are pathophysiologically based on the mechanism of atherosclerosis as influenced by dyslipidaemia, arterial hypertension, diabetes mellitus, obesity, and nicotine abuse [1].

The American Heart Association (AHA) and the American Stroke Association (ASA) have already recommended that patients with significant carotid stenosis should undergo testing for CAD [2]. The primary aim of our prospective cohort study was the other way around: to test whether findings of CAD examination predict such ICA stenoses of at least 50%, which is the earliest threshold for revascularisation in patients with stroke or transient ischemic attack (TIA) [3]. The intention was to evaluate whether a cerebrovascular examination is indicated depending on the results of the CAD diagnostics. 

The prevalence of ICA stenoses is around 4% in the general population [4] and 6% in CAD patients [5] ranging up to 30–70% in subpopulations [6,7].

Diagnostic methods for suspected CAD are chosen on the basis of the pre-test probability that a patient will reveal CAD with stenoses ≥ 50% [8]. In the guidelines, cardio-CT is suitable for patients with low to moderate CAD pre-test probabilities (15–50%). This cardio-CT examination consists of two procedures: CT calcium scoring by the Agatston equivalent score and CT coronary angiography [9,10]. It is used to assess the haemodynamic relevance of coronary artery stenoses. Due to its high negative predictive value, non-stenosing CAD can be ruled out quickly and reliably in the case of negative findings. Stenosis grading is carried out using the Coronary Artery Disease Reporting and Data System (CAD-RADS system) [11]. With a moderate pre-test probability of 15–85%, functional procedures such as stress echocardiography or myocardial perfusion Single Photon Emission Computed Tomography are used [12]. In patients with a high pre-test probability (>85%), diagnostic catheter-based coronary angiography was applied having interventional procedures for revascularization in readiness. Coronary stenoses were categorised according to AHA criteria [10].

Approximately 15–20% of ischaemic strokes are derived by cerebrovascular macroangiopathy, in particular, ICA stenoses [13,14]. Doppler and duplex ultrasonography is the gold standard for screening for stenoses and the IMT of the carotid arteries [15]. As a standard, the degree of stenosis is determined according to NASCET (North American Symptomatic Carotid Endarterectomy Trial) [16].

The primary aim of our prospective cohort study was to identify a possible association between at least 50% ICA stenosis and newly diagnosed CAD. The primary endpoint of the study is therefore to test the hypothesis that the presence of CAD on catheter-based coronary angiography or on cardio-CT predicts the presence of cerebrovascular macroangiopathy in the form of at least 50% ICA stenosis. Secondary endpoints consist of the examination of other associations between the cardiological and cerebrovascular findings. These include the correlation of the degree of ICA stenosis with the Agatston equivalent score and the value of the CAD-RADS system in cardio-CT and the degree of stenoses according to the AHA classification in catheter-based coronary angiography. The tertiary endpoints include the exploratory correlation of other cardio-CT and coronary angiography findings such as the Syntax score I with cerebrovascular findings such as the IMT of the common carotid artery (CCA).

## 2. Materials and Methods

This prospective, bicentric, cross-sectional cohort study comprised a serial patient collective of 200 adult patients who were suspected of having CAD and underwent a cardio-CT scan or a catheter-based coronary angiography as the primary diagnostic procedure at Marienhospital Gelsenkirchen (MHG) or Helios Klinikum Niederberg, both located in Northrhine Westphalia, between March 2023 and November 2023.

Based on the coronary angiography or cardio-CT findings (criterion: the presence of CAD: yes/no), the patients were divided into two groups in terms of a cohort comparison. We planned to separately recruit 100 patients per group to achieve a power of at least 80% for a two-sided alpha of 0.05. The power was calculated with G*Power Version 3.1.9.7 (Heinrich Heine University Düsseldorf, Germany).

The first study arm included patients with newly diagnosed CAD. We defined this by the presence of one or more of the following criteria: at least stenosis grade II according to the AHA classification (coronary angiography), Agatston equivalent score > 100, or CAD-RADS value > 3 (cardio-CT) [9,10,11]. However, CAD was not defined by such Agatston equivalent scores or CAD-RADS values alone, as respective patients underwent verification by coronary angiography [17]. These patients were only diagnosed with CAD if coronary angiography confirmed this diagnosis—which was the case for all of the patients with an Agatston equivalent score > 100 and CAD-RADS value > 3.

The second study arm included the patients without CAD.

Doppler and duplex sonography of the brain-supplying vessels of the recruited patients was performed by cardiologists blinded for CAD results. IMT, plaques, and the degree of stenoses were assessed.

The cut-off value of the Agatston equivalent score is specified according to age. We set the cut-off at a score of >100 [18]. The cut-off value of the CAD-RADS system is a CAD-RADS category of >3 (at least a moderate stenosis of 50–69%) [11]. The cut-off value of the AHA classification in coronary angiography is a stenosis grade of II (moderate stenosis with a longitudinal diameter of 50–75%) [10].

Inclusion criteria were an age of at least 18 years, informed written consent, and completion of both CAD and cerebrovascular examinations. Exclusion criteria encompassed pregnancy, an insufficient understanding of the German language, or a history of CAD or cerebrovascular macroangiopathy. This study was approved by the regional ethics committees.

Syntax score I describes a parameter calculated on the basis of the coronary angiography findings that is used to assess major adverse cardiac and cerebrovascular events (MACCE) [19]. Syntax score I is determined in coronary angiography from a degree of stenosis of 50% with a vessel diameter of ≥1.5 mm [20]. For the direct comparison of the Syntax score I and the resulting MACCE risk, we also determined the Syntax score I for the patient cohort without CAD, i.e., for the patients with coronary sclerosis (a degree of stenosis < 50%) in our study participants. The IMT is measured at three different points on the CCA; the average value is calculated [21].

Clinical parameters were documented for all patients: total cholesterol, low density lipoprotein (LDL) cholesterol, high-density lipoprotein (HDL) cholesterol, triglycerides, the presence of arterial hypertension, the presence of antihypertensive therapy, type II diabetes mellitus, nicotine abuse, chronic renal insufficiency, dialysis requirement, other atherosclerotic cardiovascular disease (atherosclerotic cardiovascular disease: ASCVD), a familial predisposition, and body mass index (BMI). In addition to CAD and carotid artery stenosis, ASCVD included ACS, stable angina pectoris, coronary revascularisation, aortic aneurysm, and peripheral arterial disease [22].

### Statistical Analyses

The primary endpoint of the study was the association of CAD with an ICA stenosis of at least 50%. Secondary endpoints consisted of the correlation of ICA stenosis grading with the Agatston equivalent score in cardio-CT, the value of the CAD-RADS system in coronary CT, and the degree of stenosis according to the AHA classification in catheter-based coronary angiography. For linear analyses of ICA graduations, a stenosis of 10% and higher was analysed. Tertiary/ exploratory endpoints included correlations of other cardio-CT and coronary angiography findings such as the Syntax score with cerebrovascular findings such as the presence of plaques or the IMT of the CCA and with typical risk factors. Statistical analyses were performed using IBM SPSS Statistics Version 29.0 (Statistical Package for Social Sciences, IBM, Armonk, NY, USA). Continuous variables were tested for normal distribution by the Kolmogorov–Smirnov test. We applied linear and logistic regression analyses. A significance level of two-sided alpha < 0.05 was assumed for the primary endpoint (the association of CAD and ICA stenosis of at least 50%) in multivariate analysis. The 95% confidence intervals (CI) were calculated for the binominal distribution of the primary endpoint.

## 3. Results

### 3.1. Population

In total, the data for 191 patients were fully recorded.

**Figure 1:**
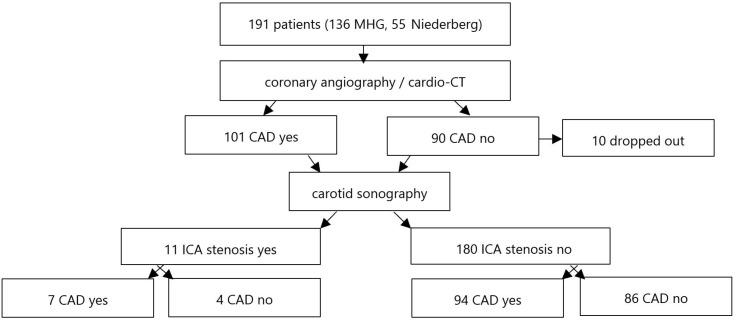
Flowchart of study design.

Due to simultaneous recruiting in two centres, 101 patients (instead of 100 as planned) were included in the CAD arm. Within the arm without CAD, 10 patients did not agree to further examinations and dropped out (Figure 1). Around 20 patients with a high pre-test probability (>85%) for CAD underwent catheter-based coronary angiography per week and 5–10 patients with a low to moderate pre-test probability (15–50%) for CAD underwent a cardio-CT examination.

A total of 55 patients were serially recruited at Helios Klinikum Niederberg, and 136 patients at Marienhospital Gelsenkirchen. The average age of the patients was 65 years (±11 years), ranging from 29 to 87 years. Of these, 111 (58%) were men and 80 (42%) were women.

Table 1 illustrates the demographic data of the patient cohorts. Table 2 summarises baseline characteristics. In total, 139 patients primarily underwent coronary angiography (*n* = 101 thereof revealed CAD), and the other 52 underwent cardio-CT (*n* = 34 with Agatston equivalent score > 100), of which 41 patients also underwent coronary CT angiography to determine the CAD-RADS value (*n* = 28 thereof with a value > 3). In total, *n* = 36 patients were positive for at least one of the two criteria. All of these 36 patients underwent subsequent coronary angiography, which in all of them confirmed CAD referring to AHA criteria, i.e., the specificity of Agatston equivalent score > 100 and CAD-RADS value > 3 was 1.0. As patients negative for CT criteria were regularly not re-examined by angiography, we cannot provide valid data on sensitivity. However, sensitivity must be below 1.0 for both criteria alone, as the proportions of respective positive patients did not perfectly match. Within the non-CAD cohort, 82 patients underwent coronary angiography, while 18 patients were diagnosed by cardio-CT. Of the 18 patients, 13 also were examined by coronary CT angiography.

### 3.2. Primary Endpoint: Association Between CAD and ICA Stenosis of at Least 50%

In the CAD (yes) group, seven of 101 patients (0.07; CI_95%_ = 0.032–0.131) had an ICA stenosis of at least 50%, whereas four of 90 patients in the CAD (no) group (0.04; CI_95%_ = 0.015–0.102) had such a stenosis (Table 3). In accordance with these overlapping CI_95%_, logistic regression (with age and gender as covariates) showed no association between CAD yes/no and ICA stenosis yes/no (Wald = 0.24; *p* = 0.624; Table 4). An additional exploratory inclusion of the risk factors total cholesterol, LDL cholesterol, HDL cholesterol, triglycerides, diabetes mellitus type II, arterial hypertension, nicotine abuse, and obesity (BMI ≥ 30 kg/m^2^) in logistic regression also revealed insignificant results (Wald = 0.004; *p* = 0.951).

In the post-hoc power analyses, the observed effect was not significant with a power of 0.73. The observed frequencies (Table 3) resulted in a sensitivity (of detecting ICA stenoses by CAD examination) of 0.64, a specificity of 0.48, a positive predictive value of 0.07, and a negative predictive value of 0.96. The prevalence of at least 50% ICA stenosis was 6% in our patient cohort: 4% in the group with CAD and 2% in the group without CAD.

### 3.3. Further Endpoints

In regression analysis, there were no significant correlations between ≥ 50% ICA stenosis and the Agatston equivalent score (Wald = 0.77; *p* = 0.448), the CAD-RADS value (Wald = 0.70; *p* = 0.490), and the AHA classification (Wald = 0.86; *p* = 0.399). Accordingly, there were no correlations of these scores with graduations of ICA stenosis from 10% up in the total population nor in the groups defined by the presence or absence of CAD, respectively. 

Interestingly, in linear regression analysis, IMT increased by age in the total population (t = 5.03; *p* < 0.001), but was not associated with the CAD parameters Agatston equivalent score (t = 0.89; *p* = 0.380), CAD-RADS score (t = 0.90; *p* = 0.377), and AHA classification (t = 0.21; *p* = 0.837) or the Syntax score I (t = 0.38; *p* = 0.706).

CAD was correlated with the presence of arterial hypertension (Wald = 4.47; *p* = 0.034) and also with type II diabetes mellitus (Wald = 7.61; *p* = 0.006), nicotine abuse (Wald = 4.83; *p* = 0.028), and BMI (Wald = 6.89; *p* = 0.009).

The Syntax score I, predicting the risk of both cardiovascular and cerebrovascular events, was independently associated with diabetes mellitus (Wald = 3.05; *p* = 0.003), sex (Wald = 3.15; *p* = 0.002), age (Wald = 2.86; *p* = 0.005), and nicotine abuse (Wald = 1.99; *p* = 0.048). However, IMT, as an indicator of cerebrovascular disease, was only associated with age (Wald = 4.36; *p* < 0.001) and nicotine abuse (Wald = 2.00; *p* = 0.047), but neither with sex (Wald = 0.41; *p* = 0.681), arterial hypertension (Wald = 1.07; *p* = 0.285), diabetes mellitus (Wald = 0.15; *p* = 0.885), BMI (Wald = 0.05; *p* = 0.958), nor with the Syntax score I itself (t = 0.38; *p* = 0.706). Similarly, an ICA stenosis of 50% was not associated with any of these covariables. 

Next, we analysed associations of ICA plaques with the covariables age, sex, total cholesterol, LDL cholesterol, HDL cholesterol, triglycerides, arterial hypertension, diabetes mellitus type II, nicotine abuse, BMI, ASCVD, and familial disposition for ASCVD. A total of 137 patients had no ICA stenosis, but ICA plaques. Of these, 75 (55%) were assigned to the cohort with CAD and 62 (45%) to the cohort without CAD. We observed significant results for age (Wald = 20.03; *p* < 0.001) and diabetes mellitus (Wald = 4.64; *p* = 0.031).

## 4. Discussion

In our study population of 191 patients, the prevalence of an ICA stenosis or other parameters of CVD did not significantly differ between patients with or without CAD.

Previous studies predominately investigated CAD in CVD patients, whereas our study’s design was vice versa. Additionally, previous studies mainly examined older patients with progressed CAD and CVD, whereas our study cohort consisted of elective patients with younger individuals without previously known CAD or CVD.

### 4.1. Comparison with Prior Studies

The analysis of the data from our cohort revealed no association between the Agatston equivalent score, the CAD-RADS score, the AHA classification or the Syntax score I with the presence or the degree of ICA stenosis according to NASCET criteria.

The large MESA study (Multi-Ethnic Study of Atherosclerosis) investigated whether carotid plaques could be a predictor of coronary artery calcification. The Agatston equivalent score was used as a coronary parameter. Carotid plaques were found to be a significant predictor of coronary artery calcification [23]. According to the MESA study, the presence and extent of carotid plaques in asymptomatic people with an Agatston equivalent score of 0 were associated with the long-term risk of CAD. With an initial Agatston equivalent score of 0 and the presence of carotid plaques, the Agatston equivalent score and the CAD risk increase over time [24]. While both our study and the MESA study involve a younger patient population with a rather low cardiovascular risk profile, our study is a cross-sectional study, whereas the MESA study has a follow-up of 16.1 years. In addition, our study only included patients with a first diagnosis of CAD (or exclusion, respectively) as a further difference to the MESA study.

In a prospective registry study of Japanese patients, no significant correlations between the degree of ICA stenosis and the maximum stenosis of the CAD were shown either, although the severity of the ICA stenosis gradually increased with the extent of the CAD [25].

In our study, Syntax score I and IMT showed no correlation (t = 0.38; *p* = 0.706). The IRAS (Insulin Resistance Atherosclerosis Study) has already shown that a rapid progression of the intima-media thickness of the carotid artery is significantly associated with the occurrence of myocardial infarction and stroke [26]. In contrast to the IRAS study, which inferred cardio- and cerebrovascular sequelae from existing carotid pathology, our study examined the presence CAD and attempted to determine whether the cardiological findings were predictive of ICA pathology.

Arterial hypertension is one of the most important risk factors for all stroke subtypes and cerebral haemorrhage. On the basis of the multicentre Framingham Study, it can be stated that the probability of a stroke increases by a factor of five in the presence of arterial hypertension [27]. In our bicentric cohort study, multiple logistic regression analysis with the variables CAD yes/no and arterial hypertension yes/no showed a significant result (Wald = 4.47; *p* = 0.034), whereas analysis with the variables ICA stenosis yes/no and arterial hypertension yes/no could not confirm an association. We defined arterial hypertension according to the 2018 ESC guideline from a blood pressure value of 140/90 mmHg [28]. Considering our statistical results alone, one could assume that cardiac pathologies such as CAD are more important for the aetiology of stroke than cerebrovascular macroangiopathies such as the at least 50% ICA stenosis. In the general population, the prevalence of ICA stenosis of approximately 1–4% is significantly lower than the prevalence of CAD of approximately 10% in total, which increases by age. Both diseases are based on the same pathophysiology, i.e., atherosclerosis. The following hypothesis could be put forward: the cardio- and cerebrovascular risk profiles appear to be quite similar, but the time until arterial hypertension has a significant effect on carotid atherosclerosis could be longer than for coronary atherosclerosis. Therefore, the early treatment of CAD might avoid or reduce the incidence of carotid stenosis. Accordingly, the ARIC study [29] and the Framingham Heart Study [30] established a significant correlation between arterial hypertension and the risk of atherosclerosis of the carotid artery. Atherosclerosis was defined here on the basis of increasing IMT and plaque deposits [29]. In line with this, a prospective study observed the coincidence of CAD and severe carotid stenosis in stroke patients over a period of approximately 5 years. The prevalence of arterial hypertension was also recorded. This was significantly higher in stroke patients with carotid stenosis and the simultaneous presence of pre-existing CAD than in patients with carotid stenosis and no CAD. The study came to the conclusion that the intensive treatment of pre-existing CAD could reduce long-term mortality and thus improve the prognosis of stroke patients with high-grade carotid stenosis [31]. Furthermore, the current literature does not provide evidence for the hypothesis that CAD and cerebrovascular disease develop with different time courses.

Diabetes increases the risk of stroke by a factor of two to four [32]. Our study found a correlation between diabetes mellitus and the presence of CAD (Wald = 7.61; *p* = 0.006) and Syntax score I (Wald = 3.05; *p*= 0.003) and ICA plaques (Wald = 4.64; *p* = 0.031). In a prospective cohort study which investigated whether carotid stenosis is predictive of the prognosis of CAD, diabetes mellitus was also found to be an important risk factor for the development of carotid stenosis [33].

### 4.2. Clinical Implications

Our study might motivate further research to explore whether CAD diagnosis can serve as an early predictor of cerebrovascular macroangiopathy. Cerebral MRI or CT brain scans including angiographies to detect both cerebrovascular macro- and microangiopathy were not available for our patient cohort and might be considered for future studies. Despite (or because of) the lack of an association between early CAD and the presence of CVD in our study, the intensive treatment of early CAD could prevent the occurrence of later CVD.

All patients in our study with an Agatston equivalent score > 100 or a CAD-RADS value > 3 in CT scans were confirmed to have CAD in coronary angiography. This emphasizes the high specificity of the CT scans for CAD diagnostics.

### 4.3. Limitations

The sample size as well as the number of clinical parameters collected are moderate, but the cohort is very homogeneous and well characterised. Data on long-term outcomes are not available. However, the aim of the study was to draw possible conclusions from the cardiovascular examination results regarding the probability of cerebrovascular macroangiopathy at a certain point in time. The cross-sectional nature of our study prohibits observations of the relevance of our findings for the clinical course of patients. The concentration on elective patients without prior CAD and CVD limits generalisability (but may provide relevant information on this selected population). The interpretation of diagnostic parameters and prognostic categories were recorded as determined by the treating cardiologist. Thus, we cannot draw any conclusions regarding intra- or interrater-variability and accuracy of diagnostic interpretation.

The post-hoc power of our primary endpoint was only 0.73 due to a generally lower frequency of ICA stenosis than expected in a priori power estimation and the drop-out of 10 patients without CAD in CT analysis who retracted agreement for CVD examination. Further studies should re-test our results.

## 5. Conclusions

The main conclusion of our study is that neither coronary angiography nor cardio- CT findings appear to be clinically sufficiently predictive of the presence of cerebrovascular macroangiopathy in elective patients without previously known vascular disease. From the clinical point of view, this suggests that the indication of CVD diagnostics may not be based on CAD findings in such patients. From the scientific point of view, these findings motivate further investigations on the time course of CAD versus CVD: speculatively, early screening and early diagnosis of CAD might help avoid or reduce CVD.

## Figures and Tables

**Table 1:** Demographic distribution of the patient cohorts.

	Helios Klinikum Niederberg (*n* = 55)	Marienhospital Gelsenkirchen (*n* = 136)	Total
CAD yes	33	68	101
Women with CAD	12	23	35
Men with CAD	21	45	66
CAD no	22	68	90
Women without CAD	11	34	45
Men without CAD	11	34	45
Average age in years	66	64	65

**Table 2:** Patient characteristics.

Median Average Age (SD)	65 Years (±11 Years)
Male	111 (58%)
Female	80 (42%)
AHA classification (coronary angiography)	181 (95%)
Agatston equivalent score (cardio-CT)	52 (27%)
CAD-RADS value (cardio-CT)	41 (22%)
Syntax Score I:	181 (95%)
Low (0–22)	169 (93%)
Intermediate (23–32)	7 (4%)
High (≥33)	5 (3%)
Grade of ICA stenosis (NASCET):	
None (0–39%)	180 (94%)
Mild (40–50%)	4 (2%)
Moderate (50–69%)	3 (2%)
Severe (≥70%)	4 (2%)
ICA plaques (<10–100%), all patients (*n* = 191)	137 (72%)
- With CAD (*n* = 101) - Without CAD (*n* = 90)	75 (74%) 62 (69%)
Risk factors:	
Total cholesterol (>200 mg/dL), all patients (*n* = 191)	66 (35%)
- With CAD (*n* = 101) - Without CAD (*n* = 90)	41 (41%) 25 (28%)
Triglycerides (>200 mg/dL), all patients (*n* = 191)	44 (23%)
- With CAD (*n* = 101) - Without CAD (*n* = 90)	24 (24%) 20 (22%)
Arterial hypertension (≥140/90 mmHg), all patients (*n* = 191)	159 (83%)
- With CAD (*n* = 101) - Without CAD (*n* = 90)	91 (91%) 68 (76%)
Diabetes mellitus Type II, all patients (*n* = 191)	67 (35%)
- With CAD (*n* = 101) - Without CAD (*n* = 90)	47 (47%) 20 (22 %)
Nicotine abuse, all patients (*n* = 191)	91 (48%)
- With CAD (*n* = 101) - Without CAD (*n* = 90)	55 (55%) 36 (40%)
Obesity (BMI ≥ 30 kg/m^2^), all patients (*n* = 191)	72 (37%)
- With CAD (*n* = 101) - Without CAD (*n* = 90)	33 (33%) 39 (43%)

**Table 3:** Four-field table: distribution of patients with and without CAD and at least 50% ICA stenosis.

	ICA Stenosis: Yes	ICA Stenosis: No	Total
CAD: yes	7 (7%)	94 (93%)	101 (100%)
CAD: no	4 (4%)	86 (96%)	90 (100%)
Total	11	180	191

**Table 4:** Logistic regression: independent association of ICA stenosis of at least 50% with CAD, age, and gender.

Dependent Variable: ICA Stenosis Yes/No	Wald	*p*
**Independent variables:**		
CAD yes/no	0.24	0.624
Age	1.70	0.192
Sex	0.54	0.462

## Data Availability

The data presented in this study are available upon request from the corresponding author. The data are not publicly available due to data protection guidelines.
